# The Effects of Fructose-Containing Sugars on Weight, Body Composition and Cardiometabolic Risk Factors When Consumed at up to the 90th Percentile Population Consumption Level for Fructose

**DOI:** 10.3390/nu6083153

**Published:** 2014-08-08

**Authors:** Joshua Lowndes, Stephanie Sinnett, Zhiping Yu, James Rippe

**Affiliations:** 1 Rippe Lifestyle Institute, 215 Celebration Place, Suite 300, Celebration, FL 34747, USA; E-Mails: jlowndes@rippelifestyle.com (J.L.); ssinnett@rippelifestyle.com (S.S.); 2 Department of Nutrition and Dietetics, University of North Florida, 1 UNF Drive, Jacksonville, FL 32224, USA; E-Mail: zhiping.nutrition@gmail.com; 3 Biomedical Sciences, University of Central Florida, Orlando, FL 32826, USA

**Keywords:** sugars, sucrose, HFCS, cardiac risk factors

## Abstract

The American Heart Association (AHA) and World Health Organization (WHO) have recommended restricting calories from added sugars at lower levels than the Institute of Medicine (IOM) recommendations, which are incorporated in the Dietary Guidelines for Americans 2010 (DGAs 2010). Sucrose (SUC) and high fructose corn syrup (HFCS) have been singled out for particular concern, because of their fructose content, which has been specifically implicated for its atherogenic potential and possible role in elevating blood pressure through uric acid-mediated endothelial dysfunction. This study explored the effects when these sugars are consumed at typical population levels up to the 90th percentile population consumption level for fructose. Three hundred fifty five overweight or obese individuals aged 20–60 years old were placed on a eucaloric diet for 10 weeks, which incorporated SUC- or HFCS-sweetened, low-fat milk at 8%, 18% or 30% of calories. There was a slight change in body weight in the entire cohort (169.1 ± 30.6 *vs*. 171.6 ± 31.8 lbs, *p* < 0.01), a decrease in HDL (52.9 ± 12.2 *vs*. 52.0 ± 13.9 mg/dL, *p* < 0.05) and an increase in triglycerides (104.1 ± 51.8 *vs*. 114.1 ± 64.7 mg/dL, *p* < 0.001). However, total cholesterol (183.5 ± 42.8 *vs*. 184.4 mg/dL, *p* > 0.05), LDL (110.3 ± 32.0 *vs*. 110.5 ± 38.9 mg/dL, *p* > 0.05), SBP (109.4 ± 10.9 *vs*. 108.3 ± 10.9 mmHg, *p* > 0.05) and DBP (72.1 ± 8.0 *vs*. 71.3 ± 8.0 mmHg, *p* > 0.05) were all unchanged. In no instance did the amount or type of sugar consumed affect the response to the intervention (interaction *p* > 0.05). These data suggest that: (1) when consumed as part of a normal diet, common fructose-containing sugars do not raise blood pressure, even when consumed at the 90th percentile population consumption level for fructose (five times the upper level recommended by the AHA and three times the upper level recommended by WHO); (2) changes in the lipid profile are mixed, but modest.

## 1. Introduction

The intake of added sugars has increased in the United States between 1970 and 2005 by 19% from 400 calories to 476 calories for Americans’ average daily energy intake [1]. Increased intakes of dietary sugar in the setting of the worldwide pandemic of cardiovascular disease (CVD) and obesity have caused concern that added sugars may be a potential causative factor in a number of serious medical conditions and diseases, such as coronary heart disease (CHD) [2], obesity [3,4,5,6,7], diabetes [8], metabolic syndrome [9] and non-alcoholic fatty liver disease (NAFLD) [10]. It has been argued that the fructose moiety in both sucrose (SUC) and high fructose corn syrup (HFCS) could be an underlying cause of these adverse consequences [11,12,13].

With these factors as the background, a number of individuals and organizations have recommended substantial reductions in the amount of added sugars in the American diet. For example, the American Heart Association (AHA) has published guidelines recommending that the average American woman should consume no more than 100 kcal per day from added sugars and the average American male no more than 150 kcal per day from added sugars [2]. These recommendations are exceeded by over 90% of individuals in the United States. The AHA recognized that these recommendations were not based on randomized clinical trials. The WHO has recommended an upper limit of 10% of calories from added sugars with an ultimate goal of a reduction to 5% of added calories [14]. Both the AHA and WHO recommendations for upper limits of added sugars are substantially lower than the 25% upper limit recommended by the Institute of Medicine (IOM) [15], which was also incorporated into the Dietary Guidelines for Americans 2010 [16].

The current, randomized controlled trial (RCT) was undertaken to explore the metabolism and impact on CHD risk factors of added sugars from the two largest sources of added sugars consumed in the American diet (SUC and HFCS) at levels up to the 90th percentile population consumption level for fructose. We explored three different levels of added sugar consumption: 8% of calories (roughly the upper level of consumption recommended by the AHA and WHO; 18% of calories (slightly above the average consumption in the United States); and 30% of calories (slightly above the upper limit recommended by the IOM and DGAs 2010). These levels represent the consumption 25th, 50th and 90th percentile population consumption levels of fructose in the American population [17]. We hypothesized that these two commonly consumed sugars at ranges of normal human consumption would not adversely impact on risk factors for CHD, that there would be no differences in these parameters between the recommendations of the AHA, WHO and IOM and that there would not be differences between SUC and HFCS related to these measures.

## 2. Material and Methods

### 2.1. Study Design

**Figure 1:**
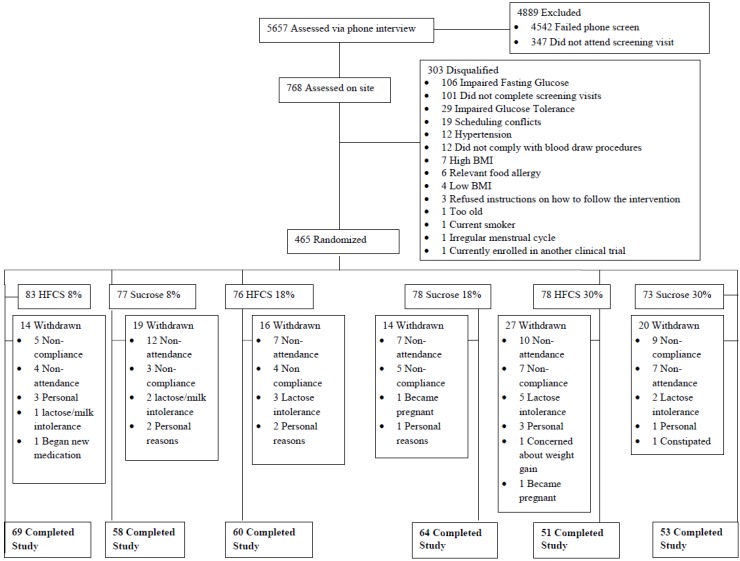
Study flow chart.

This was a randomized, prospective, parallel group, blinded study to assess the effects of consuming three different levels of SUC *vs.* HFCS as components of usual diets. Four hundred sixty five normal weight, overweight and obese subjects between the ages of 20–60 years old were randomized in the study. The present data were produced from the 355 participants who completed the intervention (see Figure 1). Both staff members and subjects were blinded as to whether or not participants in the trial were consuming SUC or HFCS. Staff members were, however, aware of whether the subjects were consuming 8%, 18% or 30% of calories as added sugar (25th, 50th and 90th percentile for fructose consumption), since this information was needed in order to prescribe the remainder of the eucaloric diet. Subjects were counseled in private counseling rooms in individual sessions to avoid the possibility of subjects talking to individuals in other groups. The duration of the study was 10 weeks for each participant. The study was approved by the Western Institutional Review Board, and all subjects signed informed consent forms.

### 2.2. Subjects

Men and women between the ages of 20–60 years old with a body mass index (BMI) of 23–35 kg/m^2^ were recruited. Individuals could not be currently enrolled in a commercial weight loss program or taking weight loss medication or supplements. Individuals were excluded if they had experienced a change in weight of greater than 3% of current body weight in the past 30 days, were currently using tobacco products or had utilized such products within the past year, had uncontrolled hypertension (≥140/90 mmHg), or were diagnosed with type 1 or type 2 diabetes, or had thyroid disease and either not taking medication or the dosage changed within the past six months, or a history of surgical procedure for weight loss, or a major surgery within three months of enrollment, or a history of heart problems (e.g., bypass surgery, myocardial infarction, *etc.*) within three months of enrollment. Individuals were also excluded if orthopedic limitations would interfere with regular physical activity, if they had a history of gastrointestinal disorders or diagnosed eating disorders, a history or the presence of cancer or congestive heart failure. Women who were pregnant, lactating or trying to become pregnant, taking any prescription medication for less than three months, or any individual consuming ≥14 alcoholic drinks per week were also excluded. A known allergy to SUC or HFCS, participation in another clinical trial within 30 days prior to enrollment, lactose intolerance or any other significant food allergy were also excluded.

Interested individuals were initially screened over the phone to determine eligibility based on self-reported data utilizing a standardized screening form and phone script to ensure that individuals were screened in a consistent manner. Self-reported data, including height and weight, were verified during the initial clinical visit. Fasting blood samples were also obtained for lipids, glucose and C-reactive protein (CRP) and uric acid (only available in a subset of 81 participants).

Each subject performed a second screening visit approximately one week later. During this visit, body composition was determined by dual X-ray absorptiometry (General Electric iDXA, Madison, WI, USA). Total lean mass, percent total body fat and percent body fat were all determined by a DXA scan. All females were required to have a negative serum pregnancy test prior to DXA testing. During this visit, research dietitians also assessed participant dietary intake by analyzing a completed 3-day food record using the Nutrient Data System for Research (NDS-R) software (University of Minnesota, Minneapolis, MN, USA). Following the 10-week intervention, repeat measurements of body composition and a repeat 3-day food record were obtained. At this time, another fasting blood sample was also obtained. All cholesterol samples were sent to a certified, research-based laboratory with error rates of less than 1%.

### 2.3. Dietary Plans

Sugar was provided in the form of 1% fat, sweetened milk (Tetra Pak, Denton, TX, USA). All participants were weight stable upon enrolling in the study, and each participant was prescribed an amount of milk, so that the added sugar contributed a certain percentage of the calories needed to maintain their initial weight. The prescriptions were as follows: 

Group 1: 8% of calories required for weight maintenance provided by added sucrose in milk (low consumption of fructose: 25th percentile of population consumption of fructose).

Group 2: 8% of calories required for weight maintenance provided by added HFCS in milk (low consumption of fructose: 25th percentile of population consumption of fructose).

Group 3: 18% of calories required for weight maintenance provided by added HFCS in milk (moderate consumption of fructose: 50th percentile of population consumption of fructose).

Group 4: 18% of calories required for weight maintenance provided by added sucrose in milk (moderate consumption of fructose: 50th percentile of population consumption of fructose).

Group 5: 30% of calories required for weight maintenance provided by added HFCS in milk (high consumption of fructose: 90th percentile of population consumption of fructose).

Group 6: 30% of calories required for weight maintenance provided by added sucrose in milk (high consumption of fructose: 90th percentile of population consumption of fructose).

The weight maintenance energy intake level was estimated using the Mifflin St. Jeor equation [18] for resting energy expenditure (REE) (with activity factor). Participants were instructed on how to account for the liquid calories in the milk, but instructed to otherwise eat their usual diet. The milk was distributed on a weekly basis, at which time, the body weight was measured and approaches to account for the liquid calories reinforced if weight gain was observed. However, no structured plan was provided to protect against potential weight gain or to counteract observed weight gain. Compliance was measured by evaluation of self-reported checklists that participants completed with each serving of milk consumed and that were handed in during the weekly milk distribution visit.

Participants were blinded to both the type and amount of sugar in the milk they consumed. Research staff were blinded to the type of sugar in each type of milk, but needed to be aware of the amount of sugar, so that the appropriate prescriptions could be made.

### 2.4. Data Analysis

Data were checked for normalcy and analyzed using a 2 (sugar type) × 3 (sugar level) analysis of variance with repeated measures (pre and post). Only data on those who completed the intervention were included in the analysis. Changes over the course of ten-weeks (Week 10 min baseline) were calculated and between group differences assessed by one**-**way ANOVA with Tukey’s *post hoc* comparisons to probe significant interaction effects. Within group changes were also tested as appropriate using paired *t*-tests without corrections for multiple comparisons. For all analyses, the alpha value was set at 0.05. All data were analyzed using SPSS Advanced Statistics V-18 (IBM, Armonk, NY, USA).

## 3. Results

### 3.1. Subjects

Of the 480 participants enrolled in the study, 355 (male = 165, female = 190) completed the 10-week intervention (26% dropout rate). On average, those who dropped out or who were withdrawn by investigators for non-compliance were similar in age and other demographic parameters compared with those who finished. A lack of compliance with the consumption of the prescribed amount of sweetened milk was the primary reason for participant attrition. Other reasons included participant unwillingness to commit to the time required, intolerance of the milk or the unwillingness to consume the amount prescribed, moving out of town, pregnancy and general dissatisfaction with the time commitment related to the study. Dropout rates were similar across all six groups.

Baseline subject data are found in Table 1.

### 3.2. Dietary Intake

Compliance to the consumption of sweetened milk in all six intervention groups was very high with >95% of all prescribed servings being consumed over the 10-week intervention period in all groups. Compliance was measured by daily food checklists, which were reviewed on a weekly basis with each subject by a research nutritionist. Sweetened milk consumption produced increases in the entire cohort in energy intake, carbohydrates, protein, total sugar and added sugar intake and a decrease in fat intake (*p* < 0.001). There was a significant time by sugar level interaction (*p* < 0.001) for all dietary components, except for fat intake. The combined 30% groups had greater increases than both the 8% and 18% groups in energy intake (650.2 ± 682.0 kcal *vs*. 15.0 ± 703.2 kcal and 325.2 ± 688.1 kcal) and protein (30.6 ± 31.7 g *vs*. 13.4 ± 31.5 g and 16.6 ± 29.7 g), whereas increases in a step-wise fashion according to sugar intake level were observed for carbohydrates (159.7 ±109.3 g *vs*. 94.1 ±91.2 g *vs*. 33.3 ± 100.4 g), total sugar (182.3 ±84.6 g *vs*. 103.2 ± 57.6 g *vs*. 55.1 ± 55.6 g) and added sugar intake (131.1 ± 69.2 g *vs*. 74.1 ± 46.1 g *vs*. 26.7 ± 49.3 g). Data for each of the six groups individually and for the entire combined cohort are presented in Table 2.

### 3.3. Body Mass and Adiposity

**Figure 2:**
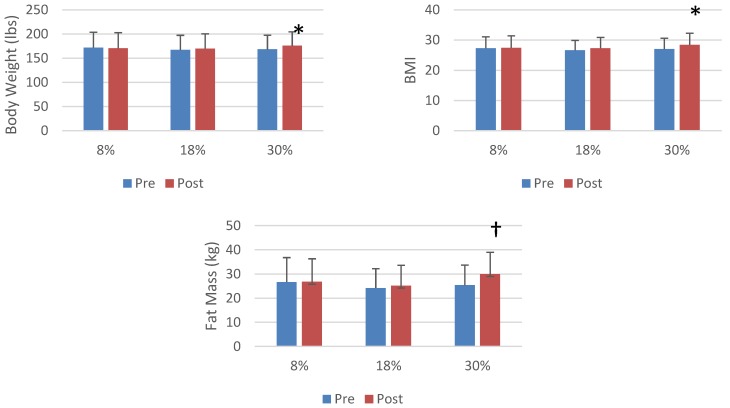
The effect of sugar consumption level on body weight and measures of adiposity. * A greater increase than 8% and 18%, *p* < 0.05. † A greater increase than 8%, *p* < 0.05.

In the entire cohort, there were significant increases in weight, BMI, percent body fat, fat mass, fat-free mass (*p* < 0.001) and waist circumference (*p* < 0.05). Data for each of the six groups and the entire combined cohort are presented in Table 3. There were significant time × sugar level interactions for body weight, BMI and fat mass (*p* < 0.05), with *post hoc* analysis revealing that the highest level of sugar intake produced greater increases in body weight and BMI than either the 8% and 18% groups and a greater increase in fat mass than in just the 8% group (Figure 2). The combined HFCS groups had a lower increase in fat-free mass (51.4 ± 10.3 kg *vs*. 51.6 ± 10.1 kg) than the combined sucrose groups (53.2 ± 11.5 kg *vs*. 53.8 kg, interaction *p* < 0.05). For no measure of weight or adiposity were the time × sugar group × sugar level interactions significant (*p* > 0.05).

### 3.4. Risk Factors for CHD

Drinking sugar-sweetened low-fat milk produced increases in the entire cohort in triglycerides (*p* < 0.001) and CRP (*p* < 0.01) and a decrease in HDL (*p* < 0.05), but there were no changes in any other risk factor (Table 4). There were no differences in the response to sugar type or sugar concentration when each was assessed independently. However, there was a significant time × sugar type × sugar level interaction for systolic blood pressure (*p* < 0.05); a statistically significant decrease was observed in the 8% sucrose group (*p* < 0.01), but no changes were observed in any of the other five groups.

**Table 1:** Baseline characteristics of the 355 participants who completed the intervention.

Baseline Characteristics	Total (*n* = 355); M = 165, F = 190	8% SUC (*n* = 58) ^1^ M = 32, F = 26	8% HFCS (*n* = 69) ^1^ M = 27, F = 42	18% HFCS (*n* = 60) M = 30, F = 30	18% SUC (*n* = 64) M = 26 F = 38	30% HFCS (*n* = 51) M = 23, F = 28	30% SUC (*n* = 53) M = 27, F = 26
Age (years)	40.19 ± 11.59	38.62 ± 12.33	38.93 ± 11.65	40.43 ± 11.33	41.30 ± 11.10	43.41 ± 11.33	38.85 ± 11.56
Weight (lbs)	169.11 ± 30.64	175.58 ± 32.93	168.63 ± 31.19	168.42 ± 30.35	165.85 ± 30.50	172.73 ± 27.58	163.90 ± 30.46
BMI	26.99 ± 3.57	27.55 ± 3.94	27.06 ± 3.73	27.06 ± 3.51	26.25 ± 3.06	27.91 ± 3.69	26.19 ± 3.27
SBP (mmHg)	109.42 ± 10.91	111.02 ± 11.22	107.65 ± 10.90	108.37 ± 9.51	107.64 ± 11.06	112.35 ± 11.28	110.43 ± 11.04
DBP (mmHg)	72.18 ± 7.97	72.00 ± 8.01	71.64 ± 6.71	72.15 ± 7.74	70.70 ± 8.87	74.66 ± 8.19	72.54 ± 8.09
Glucose (mg/dL)	89.49 ± 6.42	90.83 ± 6.15	89.39 ± 6.15	89.56 ± 6.08	88.52 ± 6.63	90.00 ± 6.74	88.77 ± 6.85

^1^ HFCS = high fructose corn syrup, SUC = Sucrose. Beverages included HFCS- or sucrose-sweetened low-fat milk to provide 8%, 18% or 30% of calories from added sugar. M = Male; F = Female.

**Table 2:** Daily dietary intake over the course of ten weeks of sugar-sweetened milk consumption.

Dietary Intake		8% Sucrose	8% HFCS ^1^	18% HFCS	18% Sucrose	30% HFCS	30% Sucrose	Pooled Cohort ^2^	Time × Sugar Type Interaction *p*	Time × Sugar Level Interaction *p*	Time × Sugar Type× Level Interaction *p*
Energy Intake (kcal)	Pre	2163.7 ± 656.5	2000.3 ± 690.3	2012.1 ± 680.6	2107.1 ± 799.8	1936.1 ± 626.2	2147.0 ± 911.0	2059.9 ± 732.2	0.236	<0.001	0.944
	Post	2230.9 ± 668.7	2171.9 ± 674.3	2364.4 ± 762.1	2406.0 ± 794.2	2641.9 ± 640.6	2743.6 ± 840.5	2411.7 ± 755.9 ***			
Fat (g)	Pre	78.4 ± 31.9	73.8 ± 30.1	77.6 ± 34.0	85.2 ± 45.8	74.6 ± 29.4	76.7 ± 37.1	77.7 ± 35.2	0.346	0.530	0.825
	Post	74.7 ±30.5	70.7 ± 30.7	72.3 ± 36.6	73.8 ±34.7	70.6 ± 26.8	68.9 ± 29.1	71.8 ± 31.5 ***			
Carbohydrate (g)	Pre	267.2 ± 86.3	241.4 ± 88.4	242.9 ± 86.2	249.7 ± 80.4	243.7 ± 92.2	275.9 ±140.1	252.9 ± 97.0	0.282	<0.001	0.671
	Post	286.6 ± 82.8	286.0 ± 85.1	339.6 ± 105.2	341.3 ± 101.9	405.8 ± 96.0	433.3 ± 138.5	345.1 ± 115.4 ***			
Protein (g)	Pre	92.3 ± 32.7	88.5 ± 43.6	86.5 ± 32.7	85.6 ± 32.5	78.1 ± 25.0	89.9 ± 41.9	87.0 ± 35.7	0.578	<0.001	0.367
	Post	105.4 ± 36.2	102.3 ± 35.9	101.4 ± 33.2	103.9 ± 37.1	112.9 ± 28.4	116.4 ± 39.7	106.7 ± 35.5 ***			
Total Sugar (g)	Pre	109.9 ± 46.8	96.8 ± 40.9	105.0 ± 49.4	106.8 ± 43.1	105.9 ± 45.1	114.1 ± 87.3	106.0 ± 53.6	0.886	<0.001	0.516
	Post	158.1 ± 49.3	157.5 ± 45.0	206.4 ± 62.6	211.7 ± 60.6	285.1 ± 69.2	299.3 ± 91.3	215.9 ± 83.8 ***			
Added Sugar (g)	Pre	71.3 ± 45.2	60.7 ± 37.6	62.6 ± 42.4	71.9 ± 42.0	73.7 ± 41.9	71.4 ± 75.7	68.2 ± 48.4	0.740	<0.001	0.288
	Post	91.0 ± 40.5	93.1 ± 39.1	138.6 ± 45.1	144.1 ± 51.9	199.4 ± 56.7	207.6 ±66.0	142.6 ±67.3 ***			

*** Different than pre, *p* < 0.001; Beverages included HFCS- or sucrose-sweetened low-fat milk to provide 8%, 18% or 30% of the calories required for weight maintenance. ^1^ HFCS = high fructose corn syrup; ^2^ “Pooled cohort” includes pooled data from all 355 participants who consumed sugar-sweetened, low-fat milk for ten weeks.

**Table 3:** Weight and adiposity over the course of ten weeks of sugar-sweetened milk consumption.

Body Composition		8% Sucrose	8% HFCS	18% HFCS	18% Sucrose	30% HFCS	30% Sucrose	Pooled Cohort	Time × Sugar Type Interaction *p*	Time × Sugar Level Interaction *p*	Time × Sugar Type × Level Interaction *p*
Body Weight (lbs)	Pre	175.6 ± 32.9	168.6 ± 31.2	168.6 ± 31.2	165.8 ± 30.5	172.7 ± 27.6	163.9 ± 30.5	169.1 ± 30.6	0.114	0.018	0.855
	Post	178.0 ± 34.1	170.5 ±32.5	169.9 ± 30.6	168.2 ± 32.7	175.8 ± 28.8	168.2 ± 31.4	171.6 ± 31.8 ***			
BMI (kg/M^2^)	Pre	27.6 ± 3.9	27.1 ±3.7	27.1 ± 3.5	26.3 ± 3.1	27.9 ± 3.7	26.2 ± 3.3	27.0 ± 3.6	0.165	0.016	0.814
	Post	27.9 ± 4.1	27.4 ± 4.0	27.3 ± 3.6	26.6 ± 3.4	28.4 ± 3.9	26.9 ± 3.3	27.4 ± 3.7 ***			
Waist Circumference (cm)	Pre	85.7 ± 10.0	84.9 ± 9.9	84.8 ± 10.1	83.1 ± 8.9	87.1 ± 9.2	83.0 ± 10.4	84.7 ± 9.8	0.054	0.268	0.943
	Post	86.0 ± 10.0	84.7 ± 10.4	84.7 ± 9.9	83.7 ± 9.9	87.5 ± 9.5	83.8 ± 10.3	85.0 ± 10.0 *			
Body Fat Percentage	Pre	32.7 ± 9.1	35.2 ± 9.0	32.6 ± 9.0	32.4 ± 8.1	37.1 ± 8.1	31.5 ± 8.5	33.6 ± 8.8	0.293	0.055	0.389
	Post	32.7 ± 9.2	35.4 ± 9.1	33.0 ± 8.8	33.2 ± 8.2	37.5 ± 8.0	32.4 ± 8.1	34.1 ± 8.7 ***			
Fat Mass (kg)	Pre	27.1 ± 11.5	26.2 ± 8.9	24.7 ± 9.0	23.7 ± 7.0	28.5 ± 8.2	22.3 ± 7.3	25.4 ± 8.9	0.778	0.022	0.128
	Post	26.4 ± 9.8	26.8 ± 9.5	25.2 ± 8.8	24.8 ± 7.5	29.6 ± 9.0	23.7 ± 7.6	26.1 ± 8.9 ***			
Fat Free Mass (kg)	Pre	55.7 ± 10.9	50.6 ± 10.9	53.0 ± 10.7	52.8 ± 12.4	50.6 ± 9.3	51.3 ± 10.9	52.3 ± 10.9	0.038	0.398	0.886
	Post	56.3 ± 11.2	50.8 ± 11.1	53.1 ± 10.1	53.2 ± 12.7	50.9 ± 9.0	52.1 ± 11.1	52.7 ± 11.0 ***			

* Different than pre *p* < 0.05; *** different than pre, *p* < 0.001. Beverages included HFCS- or sucrose-sweetened low-fat milk to provide 8%, 18% or 30% of the calories required for weight maintenance. “Pooled cohort” includes pooled data from all 355 participants who consumed sugar-sweetened, low-fat milk for ten weeks.

**Table 4:** Risk factors for cardiovascular disease over the course of ten weeks of sugar-sweetened milk consumption.

Risk Factors		8% Sucrose	8% HFCS	18% HFCS	18% Sucrose	30% HFCS	30% Sucrose	Pooled Cohort	Time × Sugar Type Interaction *p*	Time × Sugar Level Interaction *p*	Time × Sugar Type × Level Interaction *p*
Systolic Blood Pressure (mmHg)	Pre	111.0 ± 11.2	107.4 ± 10.8	108.2 ± 9.5	107.6 ± 11.1	112.4 ± 11.3	110.4 ± 11.0	109.4 ± 10.9	0.527	0.937	0.011
	Post	107.0 ± 11.1 **	108.7 ± 10.6	107.7 ± 9.2	106.3 ± 11.0	109.4 ± 12.3	111.3 ± 11.1	108.3 ± 10.9			
Diastolic Blood Pressure (mmHg)	Pre	72.0 ± 8.0	71.5 ± 6.7	72.0 ± 7.8	70.7 ± 8.9	74.7 ± 8.2	72.5 ± 8.1	72.1 ± 8.0	0.445	0.943	0.395
	Post	70.6 ± 8.7	71.0 ± 8.0	70.3 ± 7.6	71.1 ± 8.0	73.1 ± 7.4	71.8 ± 8.3	71.3 ± 8.0			
Cholesterol (mg/dL)	Pre	178.2 ± 36.8	181.2 ± 39.2	184.3 ± 40.1	188.5 ± 30.7	185.1 ± 35.8	183.5 ± 42.8	183.5 ± 37.5	0.612	0.379	0.904
	Post	176.0 + 37.0	179.6 + 38.0	185.2 + 45.7	190.9 + 35.5	187.9 + 41.6	187.9 + 47.2	184.4 + 40.8			
Triglycerides (mg/dL)	Pre	110.3 ± 6.6	97.9 ± 43.4	111.1 ± 55.3	97.3 ± 46.5	101.3 ± 48.5	108.5 ± 60.7	104.1 ± 51.8	0.595	0.136	0.193
	Post	111.9 ± 56.5	110.8 ± 8.7	109.0 ± 54.2	109.3 ± 60.3	119.3 ± 75.8	126.9 ± 83.1	114.1 ± 64.7 ***			
HDL (mg/dL)	Pre	50.8 ± 14.9	53.1 ± 139	51.0 ± 13.0	54.2 ± 16.5	51.8 ± 13.3	52.9 ± 12.2	52.4 ± 14.1	0.523	0.292	0.822
	Post	50.1 ± 13.9	52.1 ± 12.3	50.8 ± 13.5	54.3 ± 15.3	49.8 ± 12.2	52.0 ± 13.9	51.6 ± 13.6 *			
LDL (mg/dL)	Pre	105.6 ±33.8	108.4 ± 33.3	111.0 ± 33.1	114.8 ± 25.4	113.0 ± 31.0	108.9 ± 35.8	110.3 ± 32.0	0.957	0.370	0.897
	Post	103.6 ± 305	105.2 ± 31.3	112.6 ± 39.3	114.7 ± 28.5	114.5 ± 36.0	110.5 ± 38.9	110.0 ± 34.1			
Glucose (mg/dL)	Pre	90.0 ± 32.	89.1 ± 6.0	89.6 ± 90.6	88.4 ± 6.6	89.6 ± 6.7	88.8 ±6.8	89.4 ± 6.4	0.878	0.166	0.403
	Post	91.4 ± 9.6	87.9 ± 9.8	90.6 ± 7.7	88.8 ± 7.7	91.7 ± 6.7	90.2 ± 8.1	90.0 ± 8.5			
C reactive Protein (mg/L)	Pre	1.5 ± 1.6	1.9 ± 1.9	1.6 ± 1.6	2.0 ± 1.8	2.1 ± 2.1	1.5 ± 1.8	1.8 ± 1.8	0.679	0.096	0.597
	Post	2.1 ± 2.1	2.4 ± 2.3	2.0 ± 1.9	2.1 ± 2.1	2.1 ± 1.9	1.6 ± 1.3	2.1 ± 2.0 **			
Uric Acid (mg/dL)	Pre	5.3 ± 1.5	5.1 ± 1.4	6.0 ± 1.9	5.4 ± 1.3	5.0 ± 1.8	5.4 ± 1.2	5.4 ± 1.6	0.138	0.849	0.377
	Post	5.7 ± 1.2	5.1 ± 1.1	6.0 ± 2.0	5.6 ± 1.4	5.0 ± 1.5	5.6 ± 1.8	5.5 ± 1.5			

* Different than pre *p <* 0.05; ** different than pre, *p <* 0.01; *** different than pre, *p <* 0.001. Beverages included HFCS- or sucrose-sweetened low-fat milk to provide 8%, 18% or 30% of the calories required for weight maintenance. “Pooled cohort” includes pooled data from all 355 participants who consumed sugar-sweetened, low-fat milk for ten weeks.

## 4. Discussion

This blinded, randomized, prospective study compared changes in weight and body composition, lipids, blood pressure and uric acid in individuals before and after a 10-week, free living intervention during which low-fat (1%) milk was prescribed sweetened with either SUC or HFCS to deliver 8%, 18% or 30% of calories from the sweetener (25th, 50th and 90th percentile population levels for fructose consumption) in the context of mixed nutrient diets. These findings confirmed our hypothesis that consumption of sugars up to the 90th percentile population consumption level does not adversely impact cholesterol, LDL, blood pressure or glucose in the context of a well-designed, supervised, mixed nutrient diet program. To our knowledge, this is the first attempt to examine the metabolic impacts of this range of sugar and, more specifically, fructose consumption levels through normally consumed sugars amongst individuals in a free living environment.

In the current study, despite consuming levels of added sugars at the 90th percentile population consumption level for fructose consumption, individuals did not have any increase in their total cholesterol or LDL. It should be noted that these findings are different from those of some previous investigators, who have reported that added sugar consumption may increase both cholesterol and LDL [19,20,21,22]. It should also be noted that since the added sugars in the current study were delivered in low-fat milk, the increased consumption of vitamin D may have contributed to some of the results we observed [23,24]. In particular, vitamin D may contribute to LDL reduction. Thus, our reported results on cholesterol parameters must be treated with some caution. The current study also showed a 10% increase in triglycerides. It should be emphasized that both pre and post levels of triglycerides were within population established norms (104.1 ± 51.8 *vs.* 114.1 ± 64.7, *p <* 0.001). Other investigators have also reported the increased triglycerides following increased carbohydrate consumption, particularly in studies where weight gain occurs [22,25,26]. In the current study, small decreases in HDL were also observed (<1 mg/dL).

In the current study, individuals increased their caloric consumption by approximately 350 kcal/day. This resulted in weight gain and increased adiposity. Some epidemiologic studies have suggested that increased consumption of sugar-sweetened beverages may be associated with increased risk of obesity [3,4,27,28,29]. However, we have reported in previous studies that average consumption of added sugars does not increase body mass [30]. We have also reported that average consumption of either HFCS or SUC does not prevent weight loss when consumed as part of a well-designed, hypocaloric diet [31]. In the present study, the highest level of sugar intake did produce the greatest increase in weight and other measures of adiposity. This was in line with an overall greater increase in energy intake in the highest sugar intake groups, which likely provides a better explanation for the differences in weight gain rather than being due to the specific obesogenic effects of sugar. Thus, issues related to added sugars and weight gain and obesity remain undecided. Additional studies, perhaps of longer duration, will be required to clarify this area.

It has been argued that the unique metabolism of fructose, when compared with glucose, may contribute to increased levels of uric acid, leading to endothelial dysfunction and resultant increases in blood pressure [9,32,33]. Other studies have yielded different results showing no increases in blood pressure in response to added sugars [34]. However, in the current study, no increases in either systolic or diastolic blood pressure occurred at any level of added sugar consumption, nor did increases occur in uric acid. Thus, whether or not fructose consumption, even up to the 90th percentile of population consumption levels, results in either increased blood pressure or uric acid production is not supported by the current study and will require future studies for further clarification. It should also be noted that in some studies, increased calcium consumption has resulted in lower blood pressure [35,36]. Thus, our data in the area of blood pressure must be treated with some caution.

There is a significant body of evidence that suggests that there is no difference in the effect when comparing the two primary sources of fructose in the American diet, HFCS and sucrose [30,31,37,38,39,40]. In the present study, there was a greater increase in fat-free mass in the combined sucrose group than in the HFCS group, but no other differences were observed. It should also be noted that at every level of HFCS or SUC consumption up to the 90th percentile population consumption levels [17] in the current research trial, there were no differences between HFCS or SUC in any parameter measured. This provides further data to a growing body of scientific literature demonstrating that by every parameter yet measured in human beings, there are no metabolic differences between HFCS and SUC.

The strengths of the current study include its relatively large sample and the fact that it was a blinded, randomized, prospective study that explored a range of normal population consumed levels of fructose delivered through the sweeteners that are commonly consumed in the human diet, SUC and HFCS. Furthermore, levels of compliance with the intervention of drinking the sweetened milk were extremely high. The weaknesses are that the subjects were followed for only 10 weeks and that subjects over the age of 60, children and adolescents were excluded. Adolescents represent the highest fructose consuming group in the United States [17]. It should also be noted that 54% of participants in the current study were female, which may limit the ability of these data to be generalized to the public. Some animal data suggest that gender differences influence responses to fructose [41,42]. In particular, young women appear more resistant to fructose-induced hypertriglyceridemia than males. Since HFCS, sucrose and fructose consumption in the diets could not be measured, the actual differences in intake of these two sugars remain unknown, which should be taken into consideration when interpreting these data.

## 5. Conclusions

In summary, the current study showed that there were little differences over a 10-week period in free living subjects consuming average population consumption levels of fructose in the commonly consumed sugars of SUC and HFCS. What differences were present were primarily the result of the total intake of sugar and, subsequently, energy intake, rather than the identity of the sugar. Furthermore, there were no increases in blood pressure or uric acid measurements for these individuals. The expected increase in triglycerides did occur, which is consistent with both the levels of added sugars consumed, as well as the slight weight gain, which occurred over the entire cohort [43]. Importantly, while body weight and adiposity did increase in accordance with increases in sugar intake, there were no subsequent differences in metabolic parameters when comparing 8% of calories from added sugars, a level consistent with the upper limit recommended by the AHA [2] and WHO [14] with 30% of calories, which is comparable to the upper limit recommended by the IOM [15] and DGAs 2010 [16]. Our findings suggest that the issue of the appropriate upper limit for added sugar consumption is far from being settled.

The cohort increased their caloric consumption by 355 kcal and gained, on average, approximately two and one-half pounds over the 10-week trial, indicating the difficulty many individuals experienced in incorporating these levels of added sugars into their diet. Whether these findings would persist in a longer trial remains to be determined by future research.

Finally, this study adds to the expanding literature that dosage levels commonly consumed by human beings ranging from the 25th to 90th percentile of fructose consumption of HFCS and SUC behave similarly.

## Availability of Supporting Data

The data set supporting the results of this article is included within the article.
